# Lymphocytopenia and neutrophil-lymphocyte count ratio predict bacteremia better than conventional infection markers in an emergency care unit

**DOI:** 10.1186/cc9309

**Published:** 2010-10-29

**Authors:** Cornelis PC de Jager, Paul TL van Wijk, Rejiv B Mathoera, Jacqueline de Jongh-Leuvenink, Tom van der Poll, Peter C Wever

**Affiliations:** 1 Department of Emergency Medicine and Intensive Care, Jeroen Bosch Ziekenhuis, Tolbrugstraat 11, 5200 ME 's-Hertogenbosch, the Netherlands; 2 Department of Medical Microbiology and Infection Control, Jeroen Bosch Ziekenhuis, Tolbrugstraat 11, 5200 ME 's-Hertogenbosch, the Netherlands; 3 Department of Clinical Chemistry and Hematology, Jeroen Bosch Ziekenhuis, Tolbrugstraat 11, 5200 ME 's-Hertogenbosch, the Netherlands; 4 Center of Infection and Immunity Amsterdam and Center of Experimental and Molecular Medicine, University of Amsterdam, Academic Medical Center, room F4-119, meibergdreef 9, 1105 AZ Amsterdam, the Netherlands

## Abstract

**Introduction:**

Absolute lymphocytopenia has been reported as a predictor of bacteremia in medical emergencies. Likewise, the neutrophil-lymphocyte count ratio (NLCR) has been shown a simple promising method to evaluate systemic inflammation in critically ill patients.

**Methods:**

We retrospectively evaluated the ability of conventional infection markers, lymphocyte count and NLCR to predict bacteremia in adult patients admitted to the Emergency Department with suspected community-acquired bacteremia. The C-reactive protein (CRP) level, white blood cell (WBC) count, neutrophil count, lymphocyte count and NLCR were compared between patients with positive blood cultures (*n *= 92) and age-matched and gender-matched patients with negative blood cultures (*n *= 92) obtained upon Emergency Department admission.

**Results:**

Significant differences between patients with positive and negative blood cultures were detected with respect to the CRP level (mean ± standard deviation 176 ± 138 mg/l vs. 116 ± 103 mg/l; *P *= 0.042), lymphocyte count (0.8 ± 0.5 × 10^9^/l vs. 1.2 ± 0.7 × 10^9^/l; *P *< 0.0001) and NLCR (20.9 ± 13.3 vs. 13.2 ± 14.1; *P *< 0.0001) but not regarding WBC count and neutrophil count. Sensitivity, specificity, positive and negative predictive values were highest for the NLCR (77.2%, 63.0%, 67.6% and 73.4%, respectively). The area under the receiver operating characteristic curve was highest for the lymphocyte count (0.73; confidence interval: 0.66 to 0.80) and the NLCR (0.73; 0.66 to 0.81).

**Conclusions:**

In an emergency care setting, both lymphocytopenia and NLCR are better predictors of bacteremia than routine parameters like CRP level, WBC count and neutrophil count. Attention to these markers is easy to integrate in daily practice and without extra costs.

## Introduction

Bacteremia is associated with a mortality rate as high as 30% [1]. Early and accurate recognition of bacterial infections is essential for the treatment and prognosis of medical emergency admissions [2,3]. Traditional infection markers such as the white blood cell (WBC) count, neutrophil count and C-reactive protein (CRP) level are of limited value in the early detection of community-acquired bacteremia [4-6]. The search therefore continues for additional infection markers that may facilitate the prediction of bacteremia. Although new markers (for example, procalcitonin and pro-adrenomedullin) are being evaluated, the swift implementation of these markers is hampered by validation, costs and accessibility.

Absolute lymphocytopenia (lymphocyte count < 1.0 × 10^9^/l) in the course of the immune response to systemic infection is a relatively unknown phenomenon to physicians. Nevertheless, recent studies combining traditional infection markers and the lymphocyte count showed the additional value of the latter in predicting bacteremia [6-9]. Initially, lymphocytopenia has been described in case reports concerning infectious emergencies such as toxic shock syndrome [10]. Later, Zahorec demonstrated in a prospective longitudinal observational study the correlation between the severity of the clinical course and lymphocytopenia in patients treated for severe sepsis and septic shock in an oncologic intensive care unit (ICU) [7]. Hawkins and colleagues described persistent B-cell and T-cell lymphocytopenia in a cohort of 21 patients with Gram-positive and gram-negative bacteremia [9]. Also recently, Wyllie and colleagues demonstrated in two studies the clinical usefulness of lymphocytopenia in predicting bacteremia in patients with emergency medical admissions, meriting further investigation into this topic [6,8].

As the physiological immune response of circulating leucocytes to various stressful events is often characterized by an increase in neutrophil counts and a decline in lymphocyte counts, Zahorec proposed to use the ratio of the both as an additional infection marker in clinical ICU practice [7]. This so-called neutrophil-lymphocyte stress factor was found to correlate well with the severity of disease and outcome, according to Acute Physiology and Chronic Health Evaluation II and Sepsis-related Organ Failure Assessment scores [7,11,12]. Earlier, Goodman and colleagues had already shown that a so-called neutrophil:lymphocyte ratio provided a more sensitive parameter than the leucocyte count in the prediction of appendicitis [13]. Recently, Walsh and colleagues used a similar ratio - referred to as the neutrophil-to-lymphocyte ratio - as a prognostic factor in the preoperative assessment of patients with colorectal cancer [14]. In this setting, an increased neutrophil-to-lymphocyte ratio correlated with overall and cancer-specific survival. Currently, both lymphocytopenia and the neutrophil-lymphocyte count ratio (NLCR), as we refer to it, are gaining interest as independent predictors of survival in various clinical circumstances ranging from oncological patients to patients with cardiovascular diseases [15-22].

We evaluated the ability of the lymphocyte count and the NLCR, compared with traditional parameters, to predict bacteremia in patients with suspected community-acquired bacteremia upon admission to the Emergency Department (ED). As previous studies lacked an appropriate control group, we compared the CRP level, WBC, neutrophil and lymphocyte counts and the NLCR between patients with positive blood cultures and age-matched and gender-matched patients with negative blood cultures.

## Materials and methods

### Patients

Consecutive patient records from adult patients (18 years or older) admitted to the ED over a 7-month period (April to October 2005) with suspected community-acquired bacteremia were retrospectively examined. Patients were admitted to the Jeroen Bosch Hospital, an 800-bed teaching hospital in 's-Hertogenbosch, the Netherlands. The annual ED census is approximately 28,000 visits per year.

The study cohort consisted of all patients who had positive blood cultures obtained upon presentation at the ED. Patients with hematological disease, patients receiving chemotherapy and patients receiving glucocorticoids were excluded. Patients with positive blood cultures were compared with age-matched and gender-matched control patients also admitted to the ED with suspected community-acquired bacteremia but who had negative blood cultures.

Patient records from patients in both the study cohort and the control group were examined for information on previous antibiotic usage (defined as antibiotic usage on admission to the ED or within 1 week before admission) and comorbidity (chronic obstructive pulmonary disease, diabetes, renal disease, chronic liver failure, smoking and alcohol abuse). Individual patient consent was not obtained since all data used in this study were acquired retrospectively from the laboratory information system without any additional blood sampling or additional laboratory analysis. The Internal Review Board of the Jeroen Bosch Hospital ethically approves anonymous use of data retrieved from the laboratory information system.

### Microbiology

On clinical indication, blood cultures were drawn by the medical staff during the observation period in the ED. Routinely, two pairs of aerobic and anaerobic bottles were obtained and incubated for at least 5 days (BacT/ALERT; bioMérieux, Marcy l'Etoile, France). All isolates were identified by standard microbiologic procedures. Contaminated blood cultures (with, for example, coagulase-negative staphylococci or Corynebacterium species) were defined according to previously described criteria [23]. Mixed cultures were considered significant if organisms other than contaminants were isolated.

### Infection markers

CRP levels were measured with a fully automated enzyme-linked immunoassay using an Aeroset 2.0 analyzer (Abbott Diagnostics, Santa Clara, CA, USA). WBC, neutrophil and lymphocyte counts were determined on a Sysmex XE-2100 hematology analyzer (Sysmex Corporation, Kobe, Japan). The NLCR was calculated as described previously [7].

### Statistical analysis

Statistical analysis was performed using SPSS 15 (SPSS Inc, Chicago Illinois, USA). Descriptive analysis was performed for all variables. Student's *t *tests were used to evaluate the differences in CRP levels, WBC, neutrophil and lymphocyte counts and the NLCR between the study cohort and the control group. Because the outcome of blood tests was not normally distributed, a natural log transformation was calculated in order to be able to perform *t *tests. The Kolmogorov-Smirnov test was used to test for normal distribution of the transformed data. The chi-square test was used to assess the comparability of the characteristics in the study cohort and the control group. Receiver operating characteristic (ROC) curves were constructed to evaluate the sensitivity and specificity of the CRP level, the WBC, neutrophil and lymphocyte counts and the NLCR in predicting bacteremia. ROC curves displayed sensitivity versus 1 - specificity such that the area under the curve (AUC) varied from 0.5 to 1.0, with higher values indicating increased discriminatory ability. Confidence intervals on the AUC were calculated using nonparametric assumptions. To identify differences between the AUC of individual ROC curves, the method described by Hanley and McNeil was used [24]. *P *< 0.05 was considered to represent a statistically significant difference.

## Results

### Patients

Blood cultures were drawn from 746 patients. In 147 patients, microorganisms were cultured. In 29 patients, positive blood cultures were considered to be contamination. Fourteen patients were excluded because of hematological disease, use of chemotherapy or use of glucocorticoids. Twelve patients were excluded because of incomplete data. The study cohort thus consisted of 92 patients that had significant isolates cultured. Overall, 80% (599/746) of patients with suspected community-acquired bacteremia had negative blood cultures. Ninety-two age-matched and gender-matched control patients were selected. As in the study cohort, patients with hematological disease and patients using chemotherapy or glucocorticoids were not included in the control group. After clinical and microbiological assessment, an infectious diagnosis could be established in at least 85/92 (92%) of the patients in the control group. Ages in both patient groups ranged from 18 to 96 years, with a mean of 66 years.

Baseline characteristics including comorbidity are presented in Table 1. Other than alcohol abuse, there were no significant differences between the two groups. Previous antibiotic usage was almost equal in both groups. In the study cohort, eight (8.7%) patients were given antibiotics prior to the admission compared with seven (7.6%) patients in the control group. We thus found no association between antibiotic usage and bacteremia (and hence no influence on lymphocytopenia and the NLCR).

**Table 1:** Baseline characteristics upon presentation at the Emergency Department in the study cohort and control group

	Study cohort (*n *= 92)	Control group (*n *= 92)	*P *value
Age	66 (18-96)	66 (18-96)	NA
Female	48 (52.2)	48 (52.2)	NA
Previous antibiotic usage	8 (8.7)	7 (7.6)	0.788
COPD	16 (17.4)	19 (20.6)	0.573
Diabetes	21 (22.8)	17 (18.5)	0.466
Renal disease	8 (8.7)	9 (9.8)	0.799
Chronic liver failure	6 (6.5)	3 (3.3)	0.305
Smoking	9 (9.8)	12 (13.0)	0.487
Alcohol abuse	2 (2.2)	12 (13.0)	0.005

Data presented as number (percentage) of patients or mean (range). COPD, chronic obstructive pulmonary disease, NA, not applicable.

### Microbiology

The majority of isolates cultured from the study cohort were Gram-negative microorganisms (61%) with a predominance of *Escherichia coli *(*n *= 45). Roughly one-third (39%) of the isolates were Gram-positive microorganisms with a predominance of *Streptococcus pneumoniae *(*n *= 15). In seven patients, blood cultures grew more than one pathogen. Organisms isolated in the study cohort are presented in Table 2.

**Table 2:** Microorganisms (*n *= 100) isolated from the 92 patients in the study cohort

Gram-negative isolates	*n*	Gram-positive isolates	*n*
*Escherichia coli*	45	*Streptococcus pneumoniae*	15
*Klebsiella pneumoniae*	3	Non-Group A β-hemolytic streptococci	6
*Enterobacter cloacae*	2	Viridans streptococci	5
*Salmonella enterica *serotype paratyphi A	2	*Staphylococcus aureus*	5
*Pseudomonas aeruginosa*	2	*Enterococcus faecalis*	3
Anaerobic Gram-negative rod	2	Group A beta-hemolytic streptococci	1
*Klebsiella oxytoca*	1	*Abiotrophia defectiva*	1
*Proteus mirabilis*	1	*Clostridium *species	1
*Serratia marcescens*	1	*Propionibacterium *species	1
*Alcaligenes denitrificans*	1	Anaerobic Gram-positive rod	1
*Bacteroides fragilis*	1		

Total	61		39

### Infection markers

Infection markers upon presentation to the ED for the study cohort and the control group are shown in Table 3.

**Table 3:** Infection markers in the study cohort and control group

	Study cohort (*n *= 92)	Control group (*n *= 92)	*P *value
C-reactive protein level (mg/l)	176 ± 138	116 ± 103	0.042
White blood cell count (/l)	13.6 ± 6.6 × 10^9^	12.9 ± 5.2 × 10^9^	0.971
Neutrophil count (/l)	12.1 ± 6.1 × 10^9^	10.7 ± 5.1 × 10^9^	0.261
Lymphocyte count (/l)	0.8 ± 0.5 × 10^9^	1.2 ± 0.7 × 10^9^	< 0.0001
Neutrophil-lymphocyte count ratio	20.9 ± 13.3	13.2 ± 14.1	< 0.0001

Data presented as mean ± standard deviation.

At ED admission, the CRP level in the study cohort was significantly higher compared with the control group (mean ± standard deviation 176 ± 138 mg/l vs. 116 ± 103 mg/l; *P *= 0.042). A CRP level of 50 mg/l or more has been reported as highly suggestive of sepsis, while the combination of a CRP level of 50 mg/l or more with systemic inflammatory response syndrome was identified as the best model to diagnose infection at ICU admission [25,26]. In the study cohort, 69/92 patients had a CRP level of 50 mg/l or more (sensitivity 75.0%) against 58/92 patients in the control group (specificity 37.0%). Using 50 mg/l as the cut-off point, the positive predictive value (PPV) of CRP in diagnosing bacteremia was 54.3% against a negative predictive value (NPV) of 59.6%.

The WBC count in the study cohort did not differ significantly from the WBC count in the control group (13.6 ± 6.6 × 10^9^/l vs. 12.9 ± 5.2 × 10^9^/l). A WBC count below 4.0 × 10^9^/l or above 12.0 × 10^9^/l is used in the definition of systemic inflammatory response syndrome [27]. In the study cohort, 5/92 patients had a WBC count below 4.0 × 10^9^/l and 48/92 patients had a WBC count above 12.0 × 10^9^/l (sensitivity 57.6%). In the control group, there were no patients with a WBC count below 4.0 × 10^9^/l and 43/92 patients had a WBC count above 12.0 × 10^9^/l (specificity 53.3%). Using systemic inflammatory response syndrome criteria as the cut-off point of normal versus abnormal, the PPV of WBC count in diagnosing bacteremia was 55.2% against a NPV of 55.7%.

Likewise, there was no significant difference in neutrophil count between the study cohort and the control group (12.1 ± 6.1 × 10^9^/l vs. 10.7 ± 5.1 × 10^9^/l). In the study cohort, 53/92 patients had a neutrophil count above an arbitrarily set cut-off point of 10.0 × 10^9^/l (sensitivity 57.6%) against 37/92 patients in the control group (specificity 59.8%). Using this cut-off point, the PPV of neutrophil count in diagnosing bacteremia was 58.9% against a NPV of 58.5%.

The lymphocyte count in the study cohort was significantly lower compared with the control group (0.8 ± 0.5 × 10^9^/l vs. 1.2 ± 0.7 × 10^9^/l; *P *< 0.0001). In the study cohort, 68/92 patients had absolute lymphocytopenia (sensitivity 73.9%) against 39/92 patients in the control group (specificity 57.6%). Using a lymphocyte count below 1.0 × 10^9^/l as the cut-off point, the PPV of lymphocytopenia in diagnosing bacteremia was 63.6% against a NPV of 68.8%.

There was a significant difference in the NLCR between the study cohort and the control group (20.9 ± 13.3% vs. 13.2 ± 14.1; *P *< 0.0001). In our hospital, the upper limit of the normal range of the neutrophil count is set at 7.5 × 10^9^/l with a lower limit of the normal range of the lymphocyte count set at 1.0 × 10^9^/l. Arbitrarily, we used a cut-off point of 10.0 for the NLCR to calculate the sensitivity, specificity, PPV and NPV. In the study cohort, 71/92 patients had an NLCR higher than 10.0 (sensitivity 77.2%) against 34/92 patients in the control group (specificity 63.0%). The PPV of NLCR > 10.0 in diagnosing bacteremia was 67.6% against a NPV of 73.4%. The sensitivity, specificity, PPV and NPV for the aforementioned infection markers in diagnosing bacteremia are presented in Table 4.

**Table 4:** Sensitivity, specificity, positive predictive value and negative predictive value for infection markers in diagnosing bacteremia

	Sensitivity (%)	Specificity (%)	PPV (%)	NPV (%)
CRP level	75.0	37.0	54.3	59.6
WBC count	57.6	53.3	55.2	55.7
Neutrophil count	57.6	59.8	58.9	58.5
Lymphocyte count	73.9	57.6	63.6	68.8
NLCR	77.2	63.0	67.6	73.4

Sensitivity, specificity, positive predictive value (PPV) and negative predictive value (NPV) of the C-reactive protein (CRP) level (cut-off ≥50 mg/l), white blood cell (WBC) count (cut-off < 4.0 × 10^9^/l or > 12.0 × 10^9^/l), neutrophil count (cut-off > 10.0 × 10^9^/l), lymphocyte count (cut off < 1.0 × 10^9^/l) and the neutrophil-lymphocyte count ratio (NLCR) (cut-off > 10.0) in diagnosing bacteremia.

Additional analysis revealed no significant differences in any of the five infection markers when comparing patients with Gram-negative blood culture isolates versus patients with Gram-positive blood culture isolates (data not shown).

**Figure 1:**
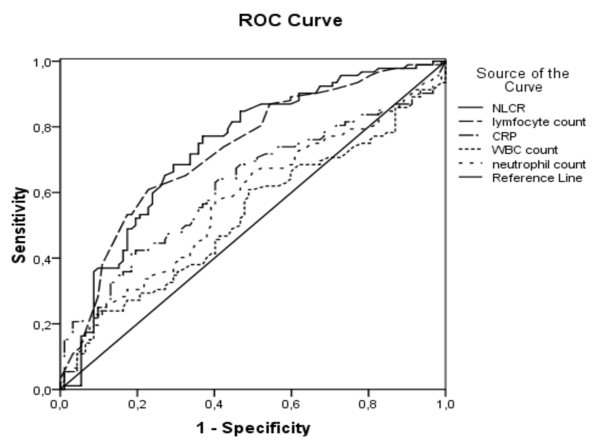
**Receiver operating characteristic curves of five infection markers for differentiating bacteremia from nonbacteremia**. Receiver operating characteristic (ROC) curves of C-reactive protein (CRP), white blood cell (WBC) count, neutrophil count, lymphocyte count and neutrophil-lymphocyte count ratio (NLCR) for differentiating bacteremia from nonbacteremia. The area under the NLCR ROC curve differed significantly from those for the CRP level, WBC count and neutrophil count. The area under the lymphocyte count ROC curve differed significantly from those for the WBC count and neutrophil count.

ROC curves of the five infection markers for differentiating bacteremia from nonbacteremia are presented in Figure 1. The AUC for the CRP level was 0.62 (confidence interval = 0.54 to 0.70). The AUC for the WBC count and for the neutrophil count was 0.53 (confidence interval = 0.44 to 0.61) and 0.57 (confidence interval = 0.49 to 0.66), respectively. The lymphocyte count and the NLCR both had the highest AUC of 0.73 (confidence interval = 0.66 to 0.80) and 0.73 (confidence interval = 0.66 to 0.81), respectively, reflecting discriminatory ability. The AUC of the NLCR ROC curve differed significantly from those for the CRP level (*P *= 0.029), WBC count (*P *< 0.01) and neutrophil count (*P *< 0.01). The AUC of the lymphocyte count ROC curve differed significantly from that for WBC (*P *< 0.01) and neutrophil count (*P *< 0.01) but not from that for the CRP level (*P *= 0.055).

## Discussion

Culturing microorganisms is the most definitive way to confirm bacterial infections. Unfortunately, this gold standard is time consuming and may be influenced by several factors including previous antibiotic usage [28,29]. Currently used conventional infection markers such as the CRP level, the WBC count and the erythrocyte sedimentation rate have relatively poor discriminatory capacity in distinguishing patients with bacterial infections versus patients with nonbacterial infections [4-6]. Increasing the diagnostic yield possibly lies in the combination of well-known parameters or the introduction of new markers.

Lymphocytopenia has previously been described as a marker of bacteremia but did not gain broad acceptance as an infection marker. The mechanisms responsible for lymphocytopenia in sepsis and septic shock involve margination and redistribution of lymphocytes within the lymphatic system and marked accelerated apoptosis [30,31]. Apoptosis is a prominent feature of sepsis [32]. This process, in which selected cell populations can be actively deleted from certain tissues, has been shown a mechanism of lymphocyte death in animal sepsis models [33-35]. Jilma and colleagues observed sustained lymphocytopenia during experimental human endotoxemia [36]. In blood of septic shock patients, lymphocyte apoptosis is rapidly increased - leading to a profound and persistent lymphocytopenia associated with poor outcome [37]. In mice, prevention of lymphocyte death in sepsis improved survival [34].

In a prospective study, Zahorec observed lymphocytopenia in 89/90 oncological ICU patients following major surgery, sepsis and septic shock. Moreover, there was a correlation between the severity of the clinical course and the extent of lymphocytopenia [7]. Later, Wyllie and colleagues highlighted the clinical usefulness of lymphocytopenia as a diagnostic marker of bacteremia in adult medical emergency admissions. On multivariate analysis, the lymphocyte count was strongly associated with bacteremia [8]. In a follow-up study, Wyllie and colleagues showed that CRP alone performed no better in bacteremia prediction than either a model combining lymphocytopenia and neutrophilia, or lymphocytopenia alone [6]. Extrapolation of these data to the emergency care unit setting is hampered, however, by the fact that in both studies admissions to the ward were included, while admission cultures were defined as those taken in the first 2 days of admission [6,8]. In our study, we exclusively investigated infection markers and blood cultures obtained during the observation period in the ED. Moreover, we used an age-matched and gender-matched control group since lymphocyte counts may gradually decline as normal adults age [38]. Our observations clearly show that lymphocytopenia performs better in predicting bacteremia in an emergency care setting than either the WBC count, neutrophil count or CRP level, with the PPVs and NPVs of lymphocytopenia outweighing predictive values of standard laboratory parameters. Absolute lymphocyte counts are readily available, making it possible to incorporate this marker in clinical decision-making. In this context, whether lymphocytopenia could add to the performance of well-accepted severity-of-illness scores would be of interest to study.

Evidence is growing that the NLCR is useful in the prediction of survival in various clinical settings. The value of the NLCR was previously explored in patients with lung cancer, patients with colorectal cancer and patients with orthotopic liver transplantation for primary hepatocellular carcinoma, and the value correlated well with overall and cancer-specific survival [14,19,21,22]. In cardiovascular medicine, the NLCR is also increasingly recognized as a predictor of prognosis. The use of the relative lymphocyte count as a prognostic parameter was soon followed by the use of the NLCR in predicting survival after coronary artery bypass grafting and chronic heart failure [15-18,20]. The NLCR is a potentially interesting parameter in predicting bacteremia in patients admitted with suspected community-acquired infections. Goodman and colleagues initially suggested the ratio's use in patients with suspected appendicitis. In their study, the NLCR was a more sensitive parameter than raised WBC count [13]. Zahorec further explored the use of the NLCR in septic oncological ICU patients and suggested that the ratio was associated with severity of disease [7]. The ability of the NLCR, compared with traditional parameters, to predict bacteremia in patients with suspected community-acquired infection in an emergency care setting has not been studied before. We show here that the AUC of the NLCR ROC curve was significantly higher than that of conventional infection markers, including the CRP level. In addition, both the PPV and NPV for predicting bacteremia were highest for the NLCR. The NLCR thus proved to be a simple infection marker with discriminatory capacity in predicting bacteremia in infectious emergency admissions.

### Limitations

As this is a derivation study the true value of lymphocytopenia and the NLCR in predicting bacteremia remains to be investigated in a prospective validation study. Although the percentage of patients with bacteremia in the entire patient group (118/746 patients, 16%) resembles data from current literature, one must consider that preselection of patients suspected with infection may have introduced an important bias. Moreover, the use of bacteremia as an outcome measure has limitations since severe nonbacteremic infections are not addressed.

There are several other causes for lymphocytopenia besides infection. For example, malnutrition may cause lymphocytopenia. Nutritional status in itself may modulate apoptosis or affect maturation through bone marrow hypoplasia [39,40]. Nutritional status was not assessed in our patients as a confounding factor.

The retrospective character of our study did not allow us to evaluate predictive values of recently developed infection markers (for example, procalcitonin, pro-adrenomedullin, neopterin) in our patients.

Positive blood cultures were used as the gold standard to establish the diagnosis of bacteremia. Nevertheless, culturing of blood is prone to errors. Especially, the volume of blood obtained for culture and the timepoint of blood sampling in relation to initiation of antimicrobial therapy are important factors [41]. Blood sampling procedures are described in local protocols but adherence to these protocols was not evaluated in this retrospective study.

## Conclusions

Absolute lymphocytopenia can be used in the prediction of infectious emergency admissions. Moreover, the ratio of neutrophil and lymphocyte counts - referred to as the NLCR - has even higher value in predicting bacteremia. This marker is simple, easily obtained and calculated, easy to integrate in daily practice and without extra costs.

## Key messages

• Absolute lymphocytopenia is a predictor of bacteremia.

• The ratio of neutrophil and lymphocyte counts has even higher value in predicting bacteremia.

• This marker is simple, easily obtained and calculated, easy to integrate into daily practice and without extra costs.

## Abbreviations

AUC: area under the curve; CRP: C-reactive protein; ED: Emergency Department; ICU: intensive care unit; NLCR: neutrophil-lymphocyte count ratio; NPV: negative predictive value; PPV: positive predictive value; ROC: receiver operating characteristic; WBC: white blood cell.

## Competing interests

The authors declare that they have no competing interests.

## Authors' contributions

CPCdeJ and PCW conceived and designed the study. CPCdeJ, PCW, PTLvW and RBM prepared the data for analysis. CPCdeJ, PTLvW and TvdP conducted the qualitative data analysis. PCW was responsible for clinical microbiological analysis of patient materials. CPCdeJ, RBM and PCW abstracted the medical records and assessed for error. JdJ-L and TvdP assisted with the interpretation of the results. CPCdeJ and PCW drafted the article and all authors contributed substantially to its revision. CPCdeJ, TvdP and PCW take responsibility for the paper as a whole.
